# Examining implicit beliefs in a replication attempt of a time-reversed priming task

**DOI:** 10.12688/f1000research.27169.2

**Published:** 2021-03-10

**Authors:** Marilyn Schlitz, Arnaud Delorme

**Affiliations:** 1 Department of Psychology, Sofia University, Palo Alto, CA, 94303, USA; 2 Department of Research, Institute of Noetic Sciences, Petaluma, CA, 94952, USA

**Keywords:** priming, Implicit Association Test

## Abstract

**Background:** Psi research is a controversial area of science that examines telepathy, clairvoyance, precognition, and psychokinesis (mind over matter). Central to the debate over the existence of psi is of whether independent investigators can replicate reportedly successful psi experiments. One important variable involves the beliefs of experimenters and participants. A preregistered experiment is presented that sought to replicate and extend previously published parapsychology experiments suggestive of precognition by examining implicit beliefs.

**Methods:** On each trial of the standard (non-psi) priming task, a pleasant or unpleasant word (the "prime") is briefly shown on computer screen, followed immediately by a pleasant or unpleasant picture. Trials on which the image and the priming word have different valences are termed “Incongruent”; trials on which the picture and the priming word share a common valence are termed “Congruent”. Participants in such experiments typically respond more slowly on Incongruent trials than on Congruent trials. In this "time-reversed" psi version of the experiment, the presumed cause-effect sequence is reversed so that the prime is not flashed until *after* the participant has already recorded his or her judgment. The experimental hypothesis remains the same: response times will be longer on trials with Incongruent prime/picture pairs than on trials with Congruent prime/picture pairs. Additionally, the study assesses expectations of success on the psi task of 32 experimenters—each testing 12 participants—using self-report questionnaires and the Implicit Association Task (IAT).

**Results:** A significant correlation was found between the Implicit Association Test (IAT) effect and the participants’ reported beliefs in psi, with the effect in the direction opposite to the hypothesized correlation.

**Conclusions: **This study offers an innovative approach to the role of beliefs in psi in a precognition study and speaks to the challenges of replication in controversial science.

## Introduction

Psi research involves the study of extended human capacities, including telepathy, clairvoyance, precognition, and psychokinesis (mind over matter). It is an area of controversial research that began in the late 1800’s and continues today. Although proponents can point to an extensive body of evidence that supports the existence of psi, most academic psychologists do not believe that psi effects are likely to exist ( Wagner & Monnet, 1979). Central to the debate is the issue of replication. Can independent investigators replicate reportedly successful psi experiments? And if so, under what conditions?

One important line of research involves the beliefs and expectations about psi. on the part of the experimenters and participants. This issue of experimenter beliefs and effects has been widely explored in mainstream psychology. For example, Rosenthal (1994, p. 176) 1 refers to 464 studies of interpersonal expectancy effects with an overall d of .63 (r = .30) that demonstrates the effect in a variety of contexts, including studies of reaction time, interpretation of inkblots, animal learning, person perception and skill learning. Harris & Rosenthal (1985) 2 provide a meta-analysis of 135 studies that focus on 16 behaviours hypothesised to mediate the effect, including warm interpersonal climate, experimenter expectancy, focused attention, and feedback.

Focusing specifically on psi research, various studies have shown participants' beliefs toward psi is predictive of successful psi performance. In a set of classic studies, Schmeidler & McConnell (1958) examined what they referred to as a “sheep-goat effect.” In these studies, “sheep” (believers) scored higher on average on psi tests than the “goats” (disbelievers). Several meta-analyses of the literature support the initial findings, noting a significant effect over 49 studies that suggest a “belief-moderated communications anomaly” over more than a 70-year span ( Lawrence, 1993; Storm *et al*., 2012).

Palmer (1972) identified four dimensions that were measured in these studies. These include two impersonal criteria that assessed belief in psi and belief that psi could be demonstrated in the experiment. The other two dimensions include personal beliefs about whether participants believe they have psi abilities and how well the participants expected to demonstrate psi in the study. In these dimensions there is a distinction between beliefs about psi and expectancies about future performance.

The current study is the third in a series that focused on the correlation of belief and outcome on a psi task ( Schlitz *et al*., 2021). In particular, the focus in the three studies was to replicate an experiment published by Daryl Bem (2011). Using a variety of protocols, Bem’s nine experiments tested for possible retroactive influences of well-established psychological effects (e.g. priming) by “time-reversing” the stimulus and response: on each trial, a participant’s response was recorded *before* the purportedly causal stimulus was presented. Bem reported statistically significant results in eight of the nine experiments, with a statistically significant mean effect size ( *d*) of 0.22 (Stouffer’s *z* = 6.66, *p* = 1.34 × 10 ^-11^). Critics argued that the analyses were partly exploratory ( Wagenmakers *et al.*, 2011) and low powered ( Schimmack, 2012), which may result in false positives. To encourage independent replications, Bem made all his experimental materials and instructions available to other investigators. As of 2016, a meta-analysis of 90 such experiments from 33 laboratories and 14 countries reported an effect size greater than 6 sigma. 

On each trial of the standard (non-psi) priming task, a pleasant or unpleasant word (the "prime") is briefly shown on computer screen, followed immediately by a pleasant or unpleasant picture drawn from the standard International Affective Picture System (IAPS) ( Lang & Greenwald, 1993). Trials on which the image and the priming word have different valences (one pleasant and one unpleasant) are termed “Incongruent trials”; trials on which the picture and the priming word share a common valence (both pleasant or both unpleasant) are termed “Congruent trials”. Participants in such experiments typically respond more slowly on Incongruent trials than on Congruent trials.

In Bem’s "time-reversed" psi version of the experiment, the presumed cause-effect sequence is reversed so that the prime is not flashed until *after* the participant has already recorded his or her judgment of the picture's valence. The experimental hypothesis remains the same: response times will be longer on trials with Incongruent prime/picture pairs than on trials with Congruent prime/picture pairs. Both of Bem's time-reversed priming experiments were successful ( Bem, 2011), and a follow-up meta-analysis of 15 such precognitive priming experiments confirmed the hypothesis with an effect size ( *d)* of 0.11, *p* = .003 ( Bem *et al*., 2015).

In the time-reversed experiment, two potential primes are pre-designated for each picture, one pleasant and one unpleasant. Immediately after the participant records his or her judgment of the picture as pleasant or unpleasant on a trial, the computer randomly selected one of the two words to serve as the priming word and flashes it briefly on the screen. This procedure thus provides a genuine sampling-with-replacement or an "open deck" procedure for determining whether a trial will be congruent or incongruent. Thus the probability of its being congruent or incongruent remains constant at .5 across all trials. As a result, there is no (non-psi) way for a participant to anticipate the kind of trial currently appearing on the screen.

The two experiments reported by Schlitz *et al*. (2021) involved three levels of participants: (1) professors and other investigators who recruited student experimenters and were invited to serve as participants themselves; (2) student experimenters who received standardized training in the experimental procedure; and (3) participants who engaged in the psi task. Investigators who conducted the experiment in a university setting obtained their own IRB approvals and were offered the option of co-authorship on the final report. All experiments were pre-registered with the Koestler Parapsychology Unit. All participants were selected based on their interest in the study, but not on their beliefs in psi. As planned, the first 32 experimenters who submitted complete data sets were used in the analysis. (The two other experimenters did not return all the necessary data sets.)

The study reported here was designed to build on the previous studies to provide a systematic attempt to explore factors that might contribute to the mixed outcomes of previous studies. In particular, it addresses a potential limitation of the first two studies in that expectancies and beliefs were evaluated using self-report questionnaires. Some studies show that when it comes to delicate social and psychological questions, including race, spirituality and core beliefs, people’s introspections are often not aligned with their instinctive responses. The current study addressed this problem by hypothesizing that implicit association tests provide a "window" into unconscious beliefs. In a first of its kind protocol, a 12-minute test was added for each experimenter and participant to assess the role of their implicit beliefs and expectancies and to see how these factors affect psi performance. The goal was to identify the extent to which people’s introspection of their belief in psi might not reflect their true core belief. This was tested using an implicit task involving word association and reaction time to assess experimenters’ and participants’ implicit belief in psi.

The Implicit Association Test (IAT), developed by Greenwald *et al*. (1998), was adapted to this study by using quick behavioral responses (such as “psi is good”, “psi is bad”) to evaluate implicit belief. The IAT is an assessment that was developed to determine the strength of a person's subconscious association between mental concepts in memory. It is typically used to assess implicit stereotypes that may be held by study participants, such as unconsciously associating stereotypically ethnic names with words consistent with ethnic stereotypes. Participants were instructed to choose if this concept applied to them or not. Their reaction time was used to assess their unconscious belief.

Over the past several decades, IAT has been used in various studies, with mixed results. As Meissner *et al.* (2019) observed, there are limitations in their predictive value for behavioral criteria and in their incremental validity over and above self-report measures is not robust. Reviews of the research suggest that the results may point to measuring associations instead of propositional beliefs, limiting their ability to predict behavior. However, they argue that recent research has addressed this problem by identifying procedures to measure implicit beliefs and increase the connections between behaviors and implicit measures. Advanced methods for analysis were incorporated into this study to strengthen the reliability of the outcomes.

## Methods

### Study design and participants

This study employed a fast-thinking protocol using retrocausal priming. 32 experimenters were recruited through online networks and forums. Once they expressed interest in participating, they were informed that they would need a computer and were responsible for recruiting 12 participants each. When they completed the data collection and returned the data to the researchers, they received a $100 Visa Gift Card and a book on psi research. When they enrolled in the study, they were sent a link to complete the Implicit Association Task (IAT) themselves. They were informed that the experiment takes about 15 minutes to complete. Once they recruited 16 people from their friends and family, they would send a link to the participants for the IAT and psi task. The participants were asked to find a quiet place where they could be free from distractions. They were contacted several times during the data collection period to check in and confirm the number of participants that had completed the experiment with them. The data were collected during a period from August, 2018 through February, 2019.

Both experimenters and participants were assessed for their baseline belief in psi phenomena using 5 simple questions to assess belief in psi (see *Extended data:* Annex 1 in file Documentation_release ( Schlitz & Delorme, 2020)). Experimenter and participant implicit belief in paranormal phenomena was assessed using an Implicit-association Test (IAT) 12-minute protocol. Each experimental session consisted of 40 trials. In each trial an image was randomly selected and displayed to the participant, followed by a randomly selected incongruent or congruent priming word.

This study was approved by the Institute of Noetic Sciences (Protocol DELA_2015). All participants and experimenters signed an informed consent before participating in the study. This form was approved by the IRB. All primary study analyses were preregistered with the Koestler Parapsychology Registry ( KPU Registry 1051).

### IAT procedure

The IAT procedure involved a series of seven tasks ( Nosek *et al*., 2007). In the first task, participants were asked to categorize words in two categories – pro psi words: “psychic," “paranormal” and “metaphysical “– versus skeptic words: “skeptic”, “materialist”,” nihilist”. Each word appeared in the middle of the screen, and the participants were asked to press the A button on the computer for the psi category and the L button for the skeptic category (with two different hands). On the second task, participants completed a similar sorting procedure with the two categories: good (A key) – words good, great, correct – versus bad (L key) – words bad, awful, wrong. On the third and fourth task (two tasks are created to give participants a break between tasks), individuals were asked to complete a combined task that included both the categories and attributes from the first two tasks. In our case, we linked categories of psi and good (A key) versus skeptic and bad (L key). The fifth task was the same as the first task with the button position for two categories psi (L key) and skeptic (A key) inverted. The sixth and seventh tasks were the same as the third and fourth task with the opposite association, skeptic and good (A key) versus psi and bad (L key). The number of trials in each task was 6, 6, 24, 24, 6, 24, and 24.

Only the data from tasks 3, 4, 6 and 7 were analyzed, as the other sessions are just for training and making sure experimenters and individuals make the correct associations. All experiments were conducted online. Experimenters sent links to perform the studies to their participants. If experimenters collected data on more than 20 participants, participants above 20 were ignore. Experimenters were compensated $100.

### Hypotheses

Hypothesis 1 states that the response time will be shorter for trials with congruent words than for trials with incongruent words. Hypotheses 2 – 4 examine the effect of participant IAT effect, experimenter IAT effect, and both participant and experimenter IAT effect, respectively. As in Bem (2011), four statistical tests were performed using two response time data transformation (1/RT and log(RT)) combined with two outlier cutoff criteria (exclude trials with response times >1500 ms and > 2500 ms). These will be referred to as iRT1500 (inverse RT with cutoff at 1500 ms), iRT2500 (inverse RT with cutoff at 2500 ms), lRT1500 (log RT with cutoff at 1500 ms), lRT2500 (log RT with cutoff at 2500 ms).

### Statistical procedure

Data cleaning followed the procedures specified in KPU registry ID number 1051. Deviations in statistical methodology from those specified in KPU registry ID number 1051 were only on account of model assumption violations that, if ignored, would have rendered the results invalid and untrustworthy. All analyses were conducted using the statistical programming language R v 3.6.3 ( R Core Team, 2020). Data visualization was completed using the R package ggplot2 ( Wickham, 2016). 

### Data processing

The main data file included participant and experimenter unique ID numbers, the reaction times for 40 trials and the responses (congruent/incongruent, correct/incorrect, photo) in character string format for 40 trials ( *Underlying data*). Since the photo name is not relevant for analysis, for the 40 response columns, the character strings were split on the colon and only the first item retained. This removed the photo information from the response columns, leaving only trial information (e.g. “Ipn1” or “XCpp0”). The data were converted from wide to long format using the R package dplyr ( Wickham *et al*., 2020) and tidyr ( Wickham & Henry, 2020) such that the resulting data frame consisted of 5 columns (Participant ID, Experimenter ID, Trial Number, Reaction Time, and Response) and 15360 rows (384 participants × 40 trials each). A new variable Type was defined based on whether the Response character string had a “C” for congruent or “I” for incongruent. Specifically, Type was a binary variable such that “C” denoted congruent trails and “I” denoted incongruent trials. A new variable Correct was defined based on whether the Response character string had a “0” or “X” vs. a “1”. Specifically, if the Response character string had either an “X” or a “0”, Correct was set to “0” (Incorrect). Otherwise, Correct was set to “1” (Correct). All string manipulations were performed using the R package stringr ( Wickham, 2019). 

Participants with judging errors on 25% or more trials (i.e. 10 or more trials) were removed. Subsequently, trials with errors in judging the image (Correct = 0) and with response times less than 250 ms (Reaction Time < 250) were excluded. Two new variables were defined, the inverse reaction time (1/RT) and the log reaction time (log(RT)). Then, two new data sets were formed, one such that only reaction times less than or equal to 1500 ms were retained and a second such that only reaction times less than or equal to 2500 ms were retained. For primary analyses, means of inverse reaction time and log reaction time were taken by participant and trial type (i.e. participant specific means were calculated for congruent and incongruent trials), yielding a data set of congruent and incongruent means for 247 participants for the 2500 ms cutoff and 355 participants for the 1500 ms cutoff.

### Data exploration and visualization

Data sets of raw observations and mean reaction times were explored through summary statistics. The mean, median, and standard deviation of mean reaction time, participant IAT Effect, and experimenter IAT effect by trial type were tabulated for data sets of mean reaction times. Individual participant-specific means were color-coded by participant IAT effect and size denoted experimenter IAT effect. The mean, median, and standard deviation of all observations by trial type were tabulated. Effect sizes were calculated using the R packages effsize ( Torchiano, 2020), effectsize ( Ben-Shachar *et al*., 2020), and WRS2 ( Mair & Wilcox, 2020). For paired *t*-tests, Cohen’s *d* was calculated with 95% confidence intervals and supplemented by the explanatory measure of effect size from the Yuen’s test on trimmed means for dependent samples. For ANOVAs, Cohen’s *f* was calculated. For bootstrapped mixed effects models, standardized effect sizes were not possible to calculate due to heteroscedasticity, so unstandardized effect sizes in the form of regression coefficients are presented in the model output with bootstrap confidence intervals. 

### Hypothesis I

***Primary analysis.*** The percentage of participants who had faster average reaction times for congruent trials compared to incongruent trials was calculated for data sets using both the 1500 ms and 2500 ms cutoffs and exact binomial tests were performed to test the alternative hypothesis that the true probability of obtaining a faster congruent trial time is greater than 0.5. 

Paired *t*-tests and bootstrapped paired *t*-tests were performed to examine differences between congruent and incongruent participant-specific mean (1) inverse and (2) log reaction times for the (a) 1500 and (b) 2500 cutoffs, yielding four sets of tests. The difference of congruent and incongruent mean transformed reaction times were examined for adherence to the normality assumption using the Shapiro-Wilk and Kolmogorov-Smirnov tests as well as graphically with histograms and QQ-plots. Subsequently paired *t*-tests were performed. Bootstrapped paired *t*-tests were performed using B = 2000 bootstrap samples and the function boot.t.test, which implements the test outlined in Ch. 16 in the R package MKinfer ( Kohl, 2019).

***Secondary analysis.*** (1) Inverse and (2) log reaction times for the (a) 1500 and (b) 2500 cutoffs were used to fit linear mixed effects models with type of trial as a factor variable and participant ID as a random effect. Subsequently, ANOVA tables could be generated. However, the models deviated greatly from model assumptions of residual normality and homogeneity of variance. Box-cox transformations were successful in bringing the residuals to normality but resulted in a diamond-shaped residuals vs. fitted plotted that are most commonly seen when the response variable is bounded or truncated. In this case, truncation at 250 ms and the cutoff generated this outcome, still a violation of homogeneity of variance. As a result, the original linear mixed effects model, fitted using the R package lme4 ( Bates *et al*., 2015), was bootstrapped using B = 2000 in the R package lmeresampler ( Loy & Steele, 2020). Given the deviations from model assumptions, cases were resampled at both the participant and observation levels. 95% normal and percentile confidence intervals were generated using the R package boot ( Canty & Ripley, 2019). 

### Hypothesis II

***Primary analysis.*** Participant-specific mean (1) inverse and (2) log reaction times for the (a) 1500 and (b) 2500 cutoffs were used to perform ANCOVAs with experimenter IAT effect as a covariate and type of trial as a factor variable. ANCOVA assumptions were examined through (1) QQ-plots, Shapiro-Wilk tests, and Kolmogorov-Smirnov tests of studentized residuals (normality), (2) the Levene test of variances by type of trial (variance homogeneity), (3) ANOVA with interaction (homogeneity of regression slopes), (4) scatterplots of the data by type of trial (linearity). The normality assumption was violated for all models and as a result, Box-Cox transformations were used to transform the dependent variable such that the normality assumption was not violated. Box-Cox transformations were generated using the R package car ( Fox & Weisberg, 2019). Estimated marginal means were calculated using the emmeans package ( Lenth, 2019).

***Secondary analysis.*** (1) Inverse and (2) log reaction times for the (a) 1500 and (b) 2500 cutoffs were used to fit linear mixed effects models with experimenter IAT effect as a covariate, type of trial as a factor variable, and participant ID as a random effect. Subsequently, ANOVA tables could be utilized to generate ANCOVA outputs. However, the models deviated greatly from model assumptions of residual normality and homogeneity of variance. Homogeneity of variance was somewhat alleviated by weighting observations by participant IAT effect, but normality was still violated. Box-cox transformations were successful in bringing the residuals to normality but resulted in a diamond-shaped residuals vs. fitted plotted that is most commonly seen when the response variable is bounded or truncated. In this case, truncation at 250 ms and the cutoff generated this outcome, still a violation of homogeneity of variance. As a result, generalized linear mixed models (GLMMs) (specifically using the Gamma family), generalized additive mixed models (GAMMs) (using both the Gaussian and Gamma families), and nonparametric linear mixed effects models were attempted without improvement (GLMMs and GAMMs did not resolve violations of assumptions and non-parametric models failed to produce a fit). As a result, the original linear mixed effects model, fitted using the R package lme4 ( Bates *et al*., 2015), was bootstrapped using B = 2000 in the R package lmeresampler ( Loy & Steele, 2020). Given the deviations from model assumptions, cases were resampled at both the participant and observation levels. 95% normal and percentile confidence intervals were generated using the R package boot ( Canty & Ripley, 2019). Interactions were included in the model.

### Hypothesis III

***Primary analysis.*** Participant-specific mean (1) inverse and (2) log reaction times for the (a) 1500 and (b) 2500 cutoffs were used to perform ANCOVAs with participant IAT effect as a covariate and type of trial as a factor variable. ANCOVA assumptions were examined through (1) QQ-plots, Shapiro-Wilk tests, and Kolmogorov-Smirnov tests of studentized residuals (normality), (2) the Levene test of variances by type of trial (variance homogeneity), (3) ANOVA with interaction (homogeneity of regression slopes), (4) scatterplots of the data by type of trial (linearity). The normality assumption was violated for all models and as a result, Box-Cox transformations were used to transform the dependent variable such that the normality assumption was not violated. Box-Cox transformations were generated using the R package car ( Fox & Weisberg, 2019). Estimated marginal means were calculated using the emmeans package ( Lenth, 2019).

***Secondary analysis.*** (1) Inverse and (2) log reaction times for the (a) 1500 and (b) 2500 cutoffs were used to fit linear mixed effects models with participant IAT effect as a covariate, type of trial as a factor variable, and participant ID as a random effect. Subsequently, ANOVA tables could be utilized to generate ANCOVA outputs. For reasons stated in the previous section, the original linear mixed effects model, fitted using the R package lme4 ( Bates *et al*., 2015), was bootstrapped using B = 2000 in the R package lmeresampler ( Loy & Steele, 2020). Given the deviations from model assumptions, cases were resampled at both the participant and observation levels. 95% normal and percentile confidence intervals were generated using the R package boot ( Canty & Ripley, 2019). Interactions were included in the model.

### Hypothesis IV

***Primary analysis.*** Participant-specific mean (1) inverse and (2) log reaction times for the (a) 1500 and (b) 2500 cutoffs were used to perform ANCOVAs with participant IAT effect and experimenter IAT effect as covariates and type of trial as a factor variable. ANCOVA assumptions were examined through (1) QQ-plots, Shapiro-Wilk tests, and Kolmogorov-Smirnov tests of studentized residuals (normality), (2) the Levene test of variances by type of trial (variance homogeneity), (3) ANOVA with interaction (homogeneity of regression slopes), and (4) scatterplots of the data by type of trial (linearity). The normality assumption was violated for all models and as a result, Box-Cox transformations were used to transform the dependent variable such that the normality assumption was not violated. Box-Cox transformations were generated using the R package car ( Fox & Weisberg, 2019). Estimated marginal means were calculated using the emmeans package ( Lenth, 2019).

***Secondary analysis.*** (1) Inverse and (2) log reaction times for the (a) 1500 and (b) 2500 cutoffs were used to fit linear mixed effects models with participant IAT and experimenter IAT effect as a covariates, type of trial as a factor variable, and participant ID as a random effect. Subsequently, ANOVA tables could be utilized to generate ANCOVA outputs. For reasons stated in the previous sections, the original linear mixed effects model, fitted using the R package lme4 ( Bates *et al*., 2015), was bootstrapped using B = 2000 in the R package lmeresampler ( Loy & Steele, 2020). Given the deviations from model assumptions, cases were resampled at both the participant and observation levels. 95% normal and percentile confidence intervals were generated using the R package boot ( Canty & Ripley, 2019). Interactions were included in the model.

## Results

Results are presented in Table 1– Table 4.

**Table 1.** Summary statistics.

Type	Trial	n	Reaction time mean (ms)	Reaction time median (ms)	Reaction time std. dev. (ms)	Participant IAT effect mean	Participant IAT effect median	Participant IAT effect std. dev.	Experimenter IAT effect mean	Experimenter IAT effect median	Experimenter IAT effect std. dev.
Participant- specific; 1500 ms cutoff	C	278	1287	1310	129.08	-391	-298.8	713	-615.7	-539	492
	I	277	1279	1307	129.87	-354	-279.5	743	-622.2	-539	499
Participant- specific; 2500 ms cutoff	C	356	1694	1719	328.74	-364	-298.5	764	-589.1	-539	482
	I	356	1689	1697	331.15	-364	-298.5	763	-589.1	-539	482
Pooled; 1500 ms cutoff	C	###	1224	1250	192.19	0	8E-04	0	7.096	7.131	0.17
	I	###	1211	1246	202.27	0	8E-04	0	7.0831	7.128	0.19
Pooled; 2500 ms cutoff	C	###	1634	1608	422.35	0	6E-04	0	7.3631	7.383	0.27
	I	###	1620	1603	423.3	0	6E-04	0	7.3536	7.38	0.28

**Table 2.** Bootstrapped linear mixed effects models for the secondary analysis of Hypothesis 2.

Type	Variable	Estimate	Normal: 95% Lower	Normal: 95% Upper	Percentile: 95% Lower	Percentile: 95% Upper
Cutoff > 1500 ms; Inverse Transformation	Intercept (Type = Congruent)	0.000794	0.000781	0.000816	0.000772	0.000807
	Experimenter IAT Effect	-1.06E-08	-3.26E-08	1.31E-08	-3.49E-08	9.90E-09
	Type = Incongruent	1.65E-05	-4.54E-06	3.80E-05	-3.37E-06	3.99E-05
	Experimenter IAT Effect: Type = Incongruent	1.14E-08	-1.19E-08	3.57E-08	-1.15E-08	3.66E-08
	Residual St. Dev.	0.000135	0.000111	0.000167	0.000108	0.000161
	Participant ID (Int.) St. Dev.	9.78E-05	7.81E-05	0.00011	8.65E-05	0.000118
Cutoff > 1500 ms; Log Transformation	Intercept (Type = Congruent)	7.15	7.13	7.17	7.14	7.18
	Experimenter IAT Effect	1.23E-05	-1.18E-05	3.39E-05	-1.02E-05	3.65E-05
	Type = Incongruent	-0.0148	-0.0342	0.00459	-0.034	0.00443
	Experimenter IAT Effect: Type = Incongruent	-1.03E-05	-3.38E-05	1.34E-05	-3.41E-05	1.40E-05
	Residual St. Dev.	0.133	0.125	0.149	0.118	0.143
	Participant ID (Int.) St. Dev.	0.104	0.0878	0.113	0.0938	0.119
Cutoff > 2500 ms; Inverse Transformation	Intercept (Type = Congruent)	0.000625	0.000605	0.000646	0.000603	0.000644
	Experimenter IAT Effect	-2.12E-08	-4.61E-08	2.88E-09	-4.51E-08	2.92E-09
	Type = Incongruent	6.70E-06	-5.62E-06	1.90E-05	-6.18E-06	1.94E-05
	Experimenter IAT Effect: Type = Incongruent	1.14E-09	-1.46E-08	1.66E-08	-1.41E-08	1.74E-08
	Residual St. Dev.	0.000137	0.000125	0.000154	0.000122	0.000151
	Participant ID (Int.) St. Dev.	0.000146	0.000132	0.000156	0.000135	0.000159
Cutoff > 2500 ms; Log Transformation	Intercept (Type = Congruent)	7.42	7.39	7.45	7.39	7.45
	Experimenter IAT Effect	3.45E-05	5.64E-07	6.87E-05	7.36E-07	6.91E-05
	Type = Incongruent	-0.00677	-0.0233	0.00944	-0.0226	0.0106
	Experimenter IAT Effect: Type = Incongruent	5.55E-07	-2.10E-05	2.20E-05	-2.15E-05	2.15E-05
	Residual St. Dev.	0.187	0.183	0.197	0.177	0.191
	Participant ID (Int.) St. Dev.	0.206	0.19	0.216	0.196	0.222

**Table 3.** Bootstrapped linear mixed effects models for the secondary analysis of Hypothesis 3.

Type	Variable	Estimate	Normal: 95% Lower	Normal: 95% Upper	Percentile: 95% Lower	Percentile: 95% Upper
Cutoff > 1500 ms; Inverse Transformation	Intercept (Type = Congruent)	0.000801	0.000793	0.000816	0.000786	0.00081
	Participant IAT Effect	1.43E-09	-1.38E-08	1.65E-08	-1.29E-08	1.74E-08
	Type = Incongruent	8.68E-06	-4.61E-06	2.16E-05	-3.33E-06	2.30E-05
	Participant IAT Effect: Type = Incongruent	-1.77E-09	-1.67E-08	1.27E-08	-1.65E-08	1.29E-08
	Residual St. Dev.	0.000135	0.000112	0.000167	0.000108	0.000162
	Participant ID (Int.) St. Dev.	9.78E-05	7.79E-05	0.000109	8.69E-05	0.000119
Cutoff > 1500 ms; Log Transformation	Intercept (Type = Congruent)	7.14	7.13	7.15	7.13	7.16
	Participant IAT Effect	-2.14E-06	-1.85E-05	1.55E-05	-2.13E-05	1.29E-05
	Type = Incongruent	-0.00733	-0.02	0.00583	-0.0208	0.00535
	Participant IAT Effect: Type = Incongruent	2.64E-06	-1.40E-05	1.88E-05	-1.32E-05	1.91E-05
	Residual St. Dev.	0.133	0.125	0.149	0.119	0.143
	Participant ID (Int.) St. Dev.	0.104	0.0879	0.112	0.0945	0.119
Cutoff > 2500 ms; Inverse Transformation	Intercept (Type = Congruent)	0.000636	0.000623	0.000651	0.000622	0.00065
	Participant IAT Effect	-2.71E-09	-1.92E-08	1.34E-08	-1.79E-08	1.44E-08
	Type = Incongruent	6.97E-06	-1.22E-06	1.53E-05	-1.13E-06	1.51E-05
	Participant IAT Effect: Type = Incongruent	2.74E-09	-5.94E-09	1.14E-08	-5.93E-09	1.17E-08
	Residual St. Dev.	0.000137	0.000125	0.000154	0.000123	0.000151
	Participant ID (Int.) St. Dev.	0.000146	0.000132	0.000156	0.000135	0.000159
Cutoff > 2500 ms: Log Transformation	Intercept (Type = Congruent)	7.4	7.38	7.42	7.38	7.42
	Experimenter IAT Effect	3.56E-06	-1.98E-05	2.84E-05	-2.28E-05	2.56E-05
	Type = Incongruent	-0.00836	-0.0193	0.00276	-0.0197	0.00238
	Participant IAT Effect: Type = Incongruent	-3.58E-06	-1.63E-05	8.82E-06	-1.61E-05	9.07E-06
	Residual St. Dev.	0.187	0.183	0.197	0.177	0.191
	Participant ID (Int.) St. Dev.	0.207	0.191	0.216	0.197	0.222

**Table 4.** Bootstrapped linear mixed effects models for the secondary analysis of Hypothesis 4.

Type	Variable	Estimate	Normal: 95% Lower	Normal: 95% Upper	Percentile: 95% Lower	Percentile: 95% Upper
Cutoff > 1500 ms; Inverse Transformation	Intercept (Type = Congruent)	0.000795	0.00078	0.000817	0.000772	0.00081
	Participant IAT effect	1.17E-09	-2.75E-08	2.93E-08	-2.24E-08	3.08E-08
	Experimenter IAT Effect	-1.10E-08	-3.31E-08	1.32E-08	-3.64E-08	1.02E-08
	Type = Incongruent	1.25E-05	-9.01E-06	3.48E-05	-9.46E-06	3.55E-05
	Participant IAT effect:Experimenter IAT effect	-6.80E-13	-2.79E-11	3.00E-11	-3.42E-11	2.48E-11
	Participant IAT Effect: Type = Incongruent	-1.30E-08	-4.09E-08	1.37E-08	-4.05E-08	1.49E-08
	Experimenter IAT Effect: Type = Incongruent	6.85E-09	-1.67E-08	3.10E-08	-1.68E-08	3.11E-08
	Participant IAT effect: Experimenter IAT Effect: Type = Incongruent	-1.67E-11	-4.74E-11	1.08E-11	-4.29E-11	1.55E-11
	Residual St. Dev.	0.000135	0.000113	0.000167	0.000108	0.000162
	Participant ID (Int.) St. Dev.	9.80E-05	7.76E-05	0.00011	8.64E-05	0.00012
Cutoff > 1500 ms; Log Transformation	Intercept (Type = Congruent)	7.15	7.13	7.17	7.13	7.18
	Participant IAT effect	-2.90E-06	-3.50E-05	3.04E-05	-3.69E-05	2.51E-05
	Experimenter IAT Effect	1.25E-05	-1.35E-05	3.48E-05	-9.53E-06	3.75E-05
	Type = Incongruent	-0.0097	-0.0313	0.0128	-0.033	0.0121
	Participant IAT effect:Experimenter IAT effect	-4.45E-10	-3.63E-08	3.17E-08	-2.92E-08	3.79E-08
	Participant IAT Effect: Type = Incongruent	1.63E-05	-1.33E-05	4.57E-05	-1.10E-05	4.74E-05
	Experimenter IAT Effect: Type = Incongruent	-4.80E-06	-2.87E-05	2.08E-05	-3.06E-05	1.91E-05
	Participant IAT effect: Experimenter IAT Effect: Type = Incongruent	2.02E-08	-1.13E-08	5.43E-08	-1.59E-08	5.10E-08
	Residual St. Dev.	0.133	0.125	0.148	0.119	0.142
	Participant ID (Int.) St. Dev.	0.104	0.0883	0.113	0.0947	0.119
Cutoff > 2500 ms; Inverse Transformation	Intercept (Type = Congruent)	0.00062	0.000599	0.00064	0.000599	0.000639
	Participant IAT effect	-2.14E-08	-4.59E-08	3.27E-09	-4.35E-08	6.22E-09
	Experimenter IAT Effect	-3.04E-08	-5.49E-08	-5.76E-09	-5.60E-08	-5.69E-09
	Type = Incongruent	8.21E-06	-4.82E-06	2.13E-05	-4.61E-06	2.20E-05
	Participant IAT effect:Experimenter IAT effect	-3.24E-11	-6.00E-11	-6.06E-13	-6.80E-11	-7.43E-12
	Participant IAT Effect: Type = Incongruent	5.73E-09	-9.41E-09	1.99E-08	-7.91E-09	2.13E-08
	Experimenter IAT Effect: Type = Incongruent	2.21E-09	-1.46E-08	1.83E-08	-1.30E-08	1.95E-08
	Participant IAT effect: Experimenter IAT Effect: Type = Incongruent	4.83E-12	-1.41E-11	2.15E-11	-1.04E-11	2.62E-11
	Residual St. Dev.	0.000137	0.000125	0.000154	0.000123	0.000151
	Participant ID (Int.) St. Dev.	0.000146	0.000131	0.000156	0.000134	0.000159
Cutoff > 2500 ms: Log Transformation	Intercept (Type = Congruent)	7.43	7.4	7.45	7.4	7.45
	Participant IAT effect	3.23E-05	-1.11E-06	6.65E-05	-3.65E-06	6.37E-05
	Experimenter IAT Effect	4.88E-05	1.38E-05	8.39E-05	1.38E-05	8.28E-05
	Type = Incongruent	-0.00933	-0.0257	0.00774	-0.0268	0.00637
	Participant IAT effect:Experimenter IAT effect	5.00E-08	5.06E-09	9.10E-08	1.51E-08	1.02E-07
	Participant IAT Effect: Type = Incongruent	-9.90E-06	-3.03E-05	1.17E-05	-3.20E-05	9.37E-06
	Experimenter IAT Effect: Type = Incongruent	-1.84E-06	-2.36E-05	2.05E-05	-2.51E-05	1.96E-05
	Participant IAT effect: Experimenter IAT Effect: Type = Incongruent	-1.00E-08	-3.50E-08	1.69E-08	-3.91E-08	1.42E-08
	Residual St. Dev.	0.187	0.183	0.197	0.177	0.191
	Participant ID (Int.) St. Dev.	0.206	0.189	0.216	0.195	0.222

### Data processing

26 participants with judging errors on 25% or more trials were removed, resulting in 14320 observations from 40 trials on 358 participants. 828 additional observations were removed because the reaction time was less than 250 ms and/or the response was incorrect (judging error occurred), yielding 13492 observations on 358 participants. The data set excluding response times >1500 ms was composed of 4449 observations on 308 participants. The data set excluding response times >2500 ms was composed of 10757 observations on 357 participants.

### Data exploration and visualization

The mean, median, and standard deviation of mean reaction time, participant IAT Effect, and experimenter IAT effect by trial type are provided in Table 1. The mean, median, and standard deviation of all observations by trial type are tabulated in Table 1. Summary statistics are comparable between congruent and incongruent trials. There do not appear to be patterns that can be explained by participant IAT effect, experimenter IAT effect, or participant ID. 

### Hypothesis I

Using the 1500 cutoff, 107 out of 246 participants (43.3%) had lower mean reaction times for congruent trials, failing to support the experimental hypothesis that the true probability of a lower mean reaction time for congruent trials is greater than 0.5 ( *p* = 0.9848; 95% CI = (0.38, 1.00)). Using the 2500 cutoff, 160 out of 355 participants (45.1%) had lower mean reaction times for congruent trials, failing to support the experimental hypothesis that the true probability of a lower mean reaction time for congruent trials is greater than 0.5 ( *p* = 0.9721; 95% CI = (0.41, 1.0)). Differences between mean transformed reaction times for congruent and incongruent trials were not normally distributed. There was no evidence to support the experimental hypothesis that mean reaction times are lower for congruent compared to incongruent trials using paired *t*-tests and bootstrapped paired *t*-tests ( *p* > 0.05) or using two-sample *t*-test and bootstrapped two-sample *t*-tests ( *p* > 0.05). There was no evidence to support the hypothesis that reaction times are lower for congruent compared to incongruent trials using bootstrapped linear mixed effects models. Across cutoffs and transformations, both the normal and percentile 95% bootstrap confidence intervals for Type = Incongruent encompassed 0, indicating that the difference in trial types was not significant at level 0.05.

### Hypothesis II

Experimenter IAT effect was significant only using the > 2500 ms cutoff. Across cutoffs and transformations, the mean transformed difference between congruent and incongruent trials was not significantly different at level 0.05 ( Table 2). There was no evidence to support the hypothesis that reaction times are lower for congruent compared to incongruent trials using bootstrapped linear mixed effects models. Across cutoffs and transformations, both the normal and percentile 95% bootstrap confidence intervals for Type = Incongruent encompassed 0, indicating that the difference in trial types was not significant at level 0.05. There was also no evidence to support an experimenter IAT effect. Across cutoffs and transformations, both the normal and percentile 95% bootstrap confidence intervals for experimenter IAT effect encompassed 0, indicating that the difference in trial types was not significant at level 0.05.

### Hypothesis III

Across cutoffs and transformations, the mean transformed difference between congruent and incongruent trials was not significantly different at level 0.05 and there was no effect due to participant IAT. There was no evidence to support the hypothesis that reaction times are lower for congruent compared to incongruent trials using bootstrapped linear mixed effects models. Across cutoffs and transformations, both the normal and percentile 95% bootstrap confidence intervals for Type = Incongruent encompassed 0, indicating that the difference in trial types was not significant at level 0.05. There was also no evidence to support a participant IAT effect. Across cutoffs and transformations, both the normal and percentile 95% bootstrap confidence intervals for participant IAT effect encompassed 0, indicating that the difference in trial types was not significant at level 0.05.

### Hypothesis IV

Across cutoffs and transformations, the mean transformed difference between congruent and incongruent trials was not significantly different at level 0.05, but there was an effect due to the interaction of participant and experimenter IAT effect and the type of trial in inverse and log transformed data with 1500 ms cutoff ( *p* < 0.05), and effects due to the interaction of participant and experimenter IAT effect in inverse and log transformed data with 2500 cutoff ( *p* < 0.05). There was no evidence to support the hypothesis that reaction times are lower for congruent compared to incongruent trials using bootstrapped linear mixed effects models. Across cutoffs and transformations, both the normal and percentile 95% bootstrap confidence intervals for Type = Incongruent encompassed 0, indicating that the difference in trial types was not significant at level 0.05. There was evidence in the 2500 cutoff data of both experimenter and participant-experimenter interaction IAT effects at level 0.05 ( Table 4)

## Discussion

This study failed to replicate the initial findings by Bem. Several differences in the protocol may account for this, including the fact that the study was done on-line and that it focused on the use of the IAT as a way of better understanding the nature of unconscious beliefs. Statistical deviations from the pre-registry included mixed-models and bootstrapping since model assumptions under the pre-registry were not upheld.

The first hypothesis stated that response times would be shorter for trials with congruent words compared to trials with incongruent words. This hypothesis was not supported at level 0.05, and in fact response times were shorter for trials with incongruent words compared to trials with congruent words. The second hypothesis stated that response time effects will be greater for experimenters with positive implicit belief about the experimental outcome. This hypothesis was only supported at the 2500 cutoff for the primary analysis and only at the 2500 cutoff for the log transformed data for the secondary analysis. The third hypothesis, that response time effects will be greater for participants with positive expectations about the experimental outcome was not supported. The fourth hypothesis involved response time effects and interaction between experimenters and participants belief in psi. For the primary hypothesis, at the 1500 cutoff, there was a significant ( *p* < 0.05) effect due to the interaction between participant IAT effect, experimenter IAT effect and type of trial. At the 2500 cutoff, there was a significant ( *p* < 0.05) experimenter IAT effect and a significant participant IAT and experimenter IAT effect. The secondary hypothesis was not supported at the 1500 cutoff. At the 2500 cutoff, there were significant effects due to experimenter IAT and the interaction between participant and experimenter IAT effect.

## Conclusions

This paper reports on a multi-researcher experiment that represents an extension of the Bem’s feeling the future paradigm. The premise of Bem’s work is that one can take a standard protocol from an established area of perceptual or cognitive psychology and reverse the elements such that success at the task is possible only if the participant is able to access information that only exists in the future; in other words, predicted condition differences constitute a test for precognition. This controversial claim has unsurprisingly provoked a vociferous reaction, but also a large number of replication attempts. Although a recent meta-analysis of these replications suggests an overall significant effect, it is clear that there is wide variation in outcome across labs.

This study illustrates the development of a progressive research program that has explored the role of belief in psi replication. Overall the study failed to replicate Bem’s original findings. None of the hypotheses were verified except for Hypothesis IV of an interaction between participant-experimenter interaction and IAT effects. This study made use of an innovative approach to testing a sheep/goat effect by introducing the IAT as a way of assessing unconscious biases. Contrary to the initial prediction, the central hypothesis shows a significant trend in the direction of slower response times in relation to coherent targets in the priming paradigm. It is notable that many failures of Bem’s suite of studies have used on-line presentation in which participants’ attention is not directly monitored; given that the current study was similarly designed, this could have been a contributory factor to the failure to replicate the initial findings.

These three studies in the series aim to build upon previous research by exploring whether the observations about beliefs in psi may play a role in the replication of anomalous results under controlled conditions. A limitation of the first two experiments ( Schlitz *et al*., 2021) is that expectancies and beliefs were evaluated using self-report questionnaires; this study sought to address this weakness. The implicit association test originally developed by Greenwald *et al*. (1998) has shown that overt responses of participants do not necessarily reflect their unconscious beliefs. Results of the current study support the utility of this approach.

Overall, this research must be seen as a failure to replicate the original Bem findings. Of course, interpretation is left to the reader. One may consider two possible ways of accounting for the results in this study and the previous two in the series. It is possible that the interpretations and meta-cultural dimensions of the experimental exchanges were unexpected variables. It is also possible that a more subtle, unanticipated and uncontrolled factor may have disrupted the production of an overall effect on the main pre-registered hypothesis. For example, the study took place in diverse settings with no consistent environment, set, or setting across sub-experiments. The background and experiences of the experimenters were uncontrolled, with the exception of the interventions. Future studies might aim to select participants and experimenters that have shown talent at performing this task.

It may also be argued that previous results by Bem and others represented chance findings or undetected subtle artifacts. In this approach, it could be said that the results reported here accurately reflect the absence of a psi effect. The magnitude of the previous findings casts this interpretation in doubt. Having said this, the methodology employed in the current study was more ambitious in scope and sophisticated in terms of the use of preregistration than Bem’s original studies.

Studies in both psychology and sociology show that people tend to interpret ambiguous evidence in alignment with their prior beliefs (see, e.g. Roe, 1999). As such, it is predicted (though not pre-registered) that proponents of psi will tend to favor the first interpretation of the data and skeptics the latter. However, the inconsistent nature of our findings does not allow for a firm acceptance or rejection of either interpretation, and the issue will only be resolved by further research. The controversy generated by research into the possible existence of psi abilities reflects the theoretical and practical importance of the questions raised by such potential abilities, and we believe this justifies the additional work needed to help resolve the type of inconsistent results reported here.

## Data availability

### Underlying data

Open Science Framework: Experimenter effect, https://doi.org/10.17605/OSF.IO/XQRC5 ( Schlitz & Delorme, 2020) (registered on 28 ^th^ October 2020, https://osf.io/6qxwy).

This project contains the following underlying data in folder ‘experiment 3’ (information about these data files is included in Documentation_release.pdf documentation available on OSF):

- data.txt
- data_additional.txt
- expertimenter_additional_info.txt
- expertimenter_info.txt
- participants_additional_info.txt
- participants_info.txt

### Extended data

Open Science Framework: Experimenter effect, https://doi.org/10.17605/OSF.IO/XQRC5 ( Schlitz & Delorme, 2020) (registered on 28 ^th^ October 2020, https://osf.io/6qxwy).

This project contains the following extended data:

- Documentation_release.pdf: contains information pertaining to the protocol for the present study (experiment 3), data file descriptions, and Annex 1 (questions about belief in psi).
- Guide to the Original RPriming Data Files.pdf: contains target pictures and primes for the present study (experiment 3).

Data are available under the terms of the Creative Commons Zero "No rights reserved" data waiver (CC0 1.0 Public domain dedication).
