# Supplementary material for: Examining implicit beliefs in a replication attempt of a time-reversed priming task
Source: F1000Res. 2021 Mar 10;10:5. Originally published 2021 Jan 6. [Version 2] doi: 10.12688/f1000research.27169.2 (PMC8030106; doi:10.12688/f1000research.27169.2)
Supplement: Peer review history

# Peer review: Reviewer response for version 2

**DOI:** 10.5256/f1000research.55015.r81238

**Published:** 2021-04-07

**Authors:** Chris Roe

**Affiliations:** 1 Department of Psychology, University of Northampton, Northampton, UK

I am content with the revisions made to the paper by Schlitz & Delorme on “Examining explicit beliefs”, and am happy to approve indexing of the revised version

Is the work clearly and accurately presented and does it cite the current literature?

Yes

If applicable, is the statistical analysis and its interpretation appropriate?

Partly

Are all the source data underlying the results available to ensure full reproducibility?

Yes

Is the study design appropriate and is the work technically sound?

Yes

Are the conclusions drawn adequately supported by the results?

Yes

Are sufficient details of methods and analysis provided to allow replication by others?

Partly

Reviewer Expertise:

NA

I confirm that I have read this submission and believe that I have an appropriate level of expertise to confirm that it is of an acceptable scientific standard.

# Peer review: Reviewer response for version 1

**DOI:** 10.5256/f1000research.30013.r76817

**Published:** 2021-02-11

**Authors:** Jeffery Martin

**Affiliations:** 1 Center for the Study of Non-Symbolic Consciousness, Newport, KY, USA; 2 Transformative Technology Laboratory, Palo Alto, CA, USA; 3 Stanford University, Stanford, CA, USA

One of the benefits of this platform is the ability to review previous reviews, and thus being able to reduce duplicate effort regarding observations. In this case, that includes a comprehensive review written by Chris Roe of the University of Northampton. I recommend starting with Roe's review prior to reading this one, as I occasionally refer to his comments.

 To Roe's observations, I would add that this is a straightforward, dense and primarily technically focused paper. It continues work by this and other teams that importantly and meaningfully furthers the work being done in this domain.

 Style-wise, the paper could probably use a bit more narrative padding and context for the average academic reader. Given that, relatively few psychology researchers are familiar with the debates around the IAT and its use, or in many cases even familiar with it at all, the paper would benefit from providing some additional context for it. It would also be helpful to have the hypotheses more clearly stated early in the paper, and to have a bit more contextual analysis and narrative at both the hypothesis and field levels, as Roe notes. 

 These style differences aside, the paper is sound from a research and technical perspective, and comprehensive. Some authors prefer a more terse and technical style that demands more of their professional readers, and I do not feel they should be penalized for this. Everything a scholar needs to both evaluate and design a reproduction of the research is present (excepting things like the actual IAT software), though the paper's terse nature can make this take some digging to piece together.

 Authors of papers involving the IAT are wise to go overboard detailing their analytical procedures. In this, these authors do not disappoint. Typically, data analysis is kept at a much more cursory, but familiar level (i.e.: ANOVA outcomes, etc. - as Roe mentions). It seems as though the authors may have meant to include a bit more of this. For example, there's a section in the paper that seems to imply that they meant to include boxplots for both datasets. Given that they are mentioned several times, this would be a helpful addition.

 For a paper dealing with IAT data, it is as or more important to demonstrate that the highly specific technical aspects of the data analysis were understood and properly implemented. These authors achieve this. As Roe also notes, many of the citations in the paper deal with methodology and data analytics. I think this is important because for the average psychology researcher to truly understand the nuances of IAT data, some of these will most likely need to be chased down and absorbed.

 The authors clearly make use of both standard and advanced statistics, but primarily report on the latter. The type and degree of analysis here is a plus for those who are able to understand it, but I am concerned that it will be a minus for those who can't. The latter could be left without an understanding of the nuances provided in the reported results. In some sense, that is just part of the divide currently present in the field between those skilled in the traditional statistics it has long depended upon, and other, often younger, scholars who also have strong expertise in the more recent advances in data analytics and what they can reveal.

 One of the benefits of the analysis from someone like Delorme, whose skills are well known to and highly regarded on both sides of this divide, is that he likely carefully thought through what he felt would best answer the hypotheses given the available data rather than just including the standard statistical fare that might actually tell less of the story. When this is the case, and other more traditional statistics are not as present, I think it is helpful for the authors to provide some detail around why they arrived at that decision.

 Most of this feedback involves differences in style, and my other comments do not rise to the level of rejection. Therefore, I accept this paper, though I encourage the authors to reflect upon these comments and make changes based on them that they feel might benefit the reader.

 Overall I find this to be a very helpful article for those interested in this field and a strong and novel contribution.

Is the work clearly and accurately presented and does it cite the current literature?

Yes

If applicable, is the statistical analysis and its interpretation appropriate?

Yes

Are all the source data underlying the results available to ensure full reproducibility?

Partly

Is the study design appropriate and is the work technically sound?

Yes

Are the conclusions drawn adequately supported by the results?

Yes

Are sufficient details of methods and analysis provided to allow replication by others?

Yes

Reviewer Expertise:

psychology, cognitive science, neuroscience

I confirm that I have read this submission and believe that I have an appropriate level of expertise to confirm that it is of an acceptable scientific standard.

# Response

**Published:** 2021-02-11

**Authors:** Marilyn Schlitz

**Affiliations:** Sofia University, USA

We have reviewed this review and will be making modifications based on the suggestions.

# Response

**Published:** 2021-03-01

**Authors:** Marilyn Schlitz

**Affiliations:** Sofia University, USA

Thank you for this thoughtful review. We have revised the paper based on the suggestions. These include the following:

 The authors have read and responded to Roe’s comments.

 To address style concerns, we provided more narrative and context regarding the contributions of the study to understanding of experimenter effects and the debate and use of IAT. Further, we moved the hypotheses up so that they might be more easily identified.

 By providing more narrative and reducing unnecessary details regarding the analyses, we have sought to reduce the paper’s “terse nature.”

 We elaborated on the critiques of IAT and addressed these in terms of how we handled them in this study. We did not include ANOVA outcomes as they were not part of our preregistered analyses. Given that the reviewer feels we have done an adequate job of detailing our analytical procedures, we have removed references to boxplots.

# Peer review: Reviewer response for version 1

**DOI:** 10.5256/f1000research.30013.r76816

**Published:** 2021-01-21

**Authors:** Chris Roe

**Affiliations:** 1 Department of Psychology, University of Northampton, Northampton, UK

This paper reports on a multi-researcher experiment that represents an extension of the Bem ‘feeling the future’ paradigm. The premise of Bem’s work is that one can take a standard protocol from an established area of perceptual or cognitive psychology and reverse the elements such that success at the task is possible only if the participant is able to access information that only exists in the future; in other words, predicted condition differences constitute a test for precognition. This controversial claim has unsurprisingly provoked a vociferous reaction, but also a large number of replication attempts. Although a recent meta-analysis of these replications suggests an overall significant effect, it is clear that there is wide variation in outcome across labs. Schlitz and Delorme’s paper represents a systematic attempt to explore factors that might contribute to that variation. This focus is framed in terms of ‘experimenter effects’, but it is important to be clear that these are not peculiar to parapsychological research. For example, Rosenthal (1994, p. 176) ^1^ refers to 464 studies of interpersonal expectancy effects with an overall d of .63 (r = .30) that demonstrates the effect in a variety of contexts, including studies of reaction time, interpretation of inkblots, animal learning, person perception and skill learning. Harris and Rosenthal (1985) ^2^ provide a meta-analysis of 135 studies that focus on 16 behaviours hypothesised to mediate the effect, including warm interpersonal climate, experimenter expectancy, focused attention, and feedback.

 Notwithstanding this, Schlitz and Delorme’s exploration in the context of claimed precognition effects is important. It is not clear, however, why priming was chosen as the method to focus on – was this especially susceptible to more polarised outcomes in the meta-analyses? Was there more scope with this method for experimenter effects to gain purchase? Belief is clearly an important factor that can drive experimenter effects, but there are other factors linked especially to rapport and social comfort, and it would be useful to adopt a more multivariate approach to interpersonal dynamics in future studies. Having said that, the use of an implicit association test was a clever way to avoid social desirability confounds when looking at belief and expectation per se.

 It would have been helpful to know what other instructions were given to participant-experimenters regarding the conditions under which trials should be run; for example, whether participants were monitored during their trial, whether they were required to use a quiet space in which they would not be disturbed, whether minimum requirements for internet bandwidth and stability were in place (it is not stated that the experiment takes place online, but is implied by the description that the authors ”send a link to the participants for the IAT and psi task).

 Were the criteria used in data cleaning specified in the pre-registration? It would be important to say so – it’s a bit too open ended to simply state “It was the goal to follow the statistical methodology of KPU registry ID number 1051 as closely as possible”. The analysis strategy seems exhaustive and transparent. Having said that, I found the report of outcomes rather opaque with a number of references to outcomes supporting the null hypothesis rather than the conventional formulation that they failed to support the experimental hypothesis, and in places, the null was treated as the experimental (“There was no evidence to support the null hypothesis that reaction times are lower for congruent compared to incongruent trials”, bottom p 9). This elucidation could be usefully simplified. Reporting of H2-H4 seemed similarly unfocused. Given the analysis strategy, I expected IAT analyses to be reported in terms of ANOVA outcomes, with one factor being incongruent/congruent condition and a second factor being belief group using something like a median split; in which case, the belief effect would show up in the interaction.

 The discussion gets rather bogged down in technical issues regarding the distributions of raw data, and lacks a clear statement concerning how the findings relate to the issue we began with, namely accounting for variations in outcomes across Bem replications in terms of belief and motivation factors. As a consequence, the citations in this section relate only to statistical matters, at the expense of, say, relating current findings to previous successful and unsuccessful replication attempts that have used the priming task (e.g. Rabeyron 2014). ^3^

 It is proper to acknowledge that overall the experiment has failed to replicate Bem’s original finding. It would be interesting to speculate on design features that might have contributed to that; it is notable that many failures of Bem’s suite of studies have used on-line presentation in which participants’ attention is not directly monitored, and if the current study was similarly designed then this could have been a contributory factor. The opening paragraphs of the conclusions are more like summaries of design features than conclusions per se.

Is the work clearly and accurately presented and does it cite the current literature?

Yes

If applicable, is the statistical analysis and its interpretation appropriate?

Partly

Are all the source data underlying the results available to ensure full reproducibility?

Yes

Is the study design appropriate and is the work technically sound?

Yes

Are the conclusions drawn adequately supported by the results?

Yes

Are sufficient details of methods and analysis provided to allow replication by others?

Partly

Reviewer Expertise:

Parapsychology, Transpersonal Psychology, Anomalous Experience

I confirm that I have read this submission and believe that I have an appropriate level of expertise to confirm that it is of an acceptable scientific standard, however I have significant reservations, as outlined above.

## References

[1] : Interpersonal Expectancy Effects: A 30-Year Perspective. *Current Directions in Psychological Science*.1994;3(6) : 10.1111/1467-8721.ep10770698 176-179 10.1111/1467-8721.ep10770698

[2] : Mediation of interpersonal expectancy effects: 31 meta-analyses. *Psychological Bulletin*.1985;97(3) : 10.1037/0033-2909.97.3.363 363-386 10.1037/0033-2909.97.3.363

[3] : Retro-priming, priming, and double testing: psi and replication in a test-retest design. *Front Hum Neurosci*.2014;8: 10.3389/fnhum.2014.00154 154 10.3389/fnhum.2014.00154 24672466PMC3957021

# Response

**Published:** 2021-02-11

**Authors:** Marilyn Schlitz

**Affiliations:** Sofia University, USA

As author I have read this review and will modify the paper to address the concerns raised.

# Response

**Published:** 2021-03-01

**Authors:** Marilyn Schlitz

**Affiliations:** Sofia University, USA

R1

 The authors appreciate the thoughtful review and have addressed the feedback throughout the paper. These changes are noted below.

 We have expanded the narrative to include the topic of experimenter effects in psychology and have included references to Rosenthal and Harris and Rosental in order to better position this study within the mainstream psychology approach to expectancy effects and to reference existing meta-analyses.

*Notwithstanding this, Schlitz and Delorme’s exploration in the context of claimed precognition effects is important. It is not clear, however, why priming was chosen as the method to focus on – was this especially susceptible to more polarised outcomes in the meta-analyses? Was there more scope with this method for experimenter effects to gain purchase?*

 We make note of the fact that a multivariate approach to interpersonal dynamics would be a useful next step in future studies. We appreciate that the reviewer makes not of the innovative nature of the implicit association test to study social desirability confounds in looking at belief and expectations.

 We elaborated on the instructions that were given to the participants, including that the trials should be conducted in a quiet space and that the task took place online. All analyses, including data cleaning specifications, were included in the preplanned analyses. Further, we simplified the statistical discussion. The ANOVA analysis was not included as it had not be pre-registered. The narrative regarding the findings and how they relate to Bem’s previous research have been expanded. Likewise, we explicitly noted that the replication was unsuccessful and speculate in the Conclusion on how design features of this study, including the fact that it was conducted online, have been added.
